# Surveillance of Hospital Contacts among Danish Seafarers and Fishermen with Focus on Skin and Infectious Diseases—A Population-Based Cohort Study

**DOI:** 10.3390/ijerph111111931

**Published:** 2014-11-18

**Authors:** Linda Kaerlev, Anker Jensen, Harald Hannerz

**Affiliations:** 1 Research Unit of Clinical Epidemiology, Institute of Clinical Research, University of Southern Denmark, Odense 5000, Denmark; E-Mail: Linda.kaerlev@rsyd.dk; 2 Center for Clinical Epidemiology, Odense University Hospital, Odense 5000, Denmark; 3 Department of Occupational Health, Hospital of South Western Jutland, Esbjerg 6700, Denmark; E-Mail: anker.jensen@dadlnet.dk; 4 National Institute of Occupational Health, Copenhagen 2100, Denmark; E-Mail: hha@arbejdsmiljoforskning.dk

**Keywords:** seamen, fishermen, hospital, epidemiology, occupational, disease, surveillance, risk, skin, infection

## Abstract

*Objectives*: A systematic overview of time trends in hospital contacts among Danish seafarers and fishermen by job title and analyses on skin and infectious diseases. *Methods:* Occupational cohorts with hospital contacts 1994–1998 and 1999–2003. Standardized hospital contact ratios (SHCR) were estimated using national rates and ranked by SHCR size. *Results:* For non-officers in 1994–1998, infectious diseases had the highest SHCR, followed by neoplastic and endocrinal diseases; in 1999–2003 skin diseases were followed by endocrinal and gastrointestinal diseases. For fishermen in 1994–1998, nervous system, gastrointestinal, and skin diseases had the highest SHCRs; in 1999–2003 it was nervous system, skin, and lymphohematopoietic diseases. As for skin diseases, male fishermen and non-officer seamen generally had increased SHCRs, but engine room personnel specifically had a low SHCR for eczema (eight cases). Fishermen had high SHCRs for tuberculosis in both time periods (six and nine cases, respectively). Non-officer seamen on cargo ships had increased SHCRs for HIV in both time periods and for hepatitis in 1994–1999. Extending the follow-up until 2000 or 2005 showed similar results. *Conclusions:* Surveillance of seamen’s health gives useful information. The elevated SHCR for HIV infection among non-officers has not declined despite preventive information campaigns. Tuberculosis among fishermen may be due to infection on shore. Skin diseases had very high SHCRs, not due to cutaneous oil exposure.

## 1. Introduction

Seafarers and fishermen were in previous studies found to have working conditions with a high risk of accidents and morbidity [1,2,3,4,5,6,7,8,9,10,11,12,13,14,15,16,17,18,19,20,21]. The Danish merchant fleet has some of the most up-to-date ships in the world. The number of seamen on board with Danish nationality has declined during the last 20 years due to fewer Danish seafarers working as non-officers and an increasing number of non-officers of other nationalities on Danish ships. In the fishing fleet, fewer and larger fishing vessels dominate the trade nowadays, and the physical working environment has improved. The present burden of a large number of diseases has been suggested to be a result of working conditions that no longer exist [22,23].

Two studies from 1996, one from Denmark and one from Poland, have shown increased risk of infectious diseases among seafarers [24,25,26]. Among persons working with oil or oil products or with exposure to wet environment, an increased risk of eczema has been reported [27]. Fishermen are known to be exposed to wet environments, and engine room crew may have cutaneous oil exposure and exposure to polycyclic aromatic hydrocarbons (PAHs), but few studies have been conducted among seafarers and fishermen regarding diseases resulting from these exposures [27,28].

Using a unique combination of national registries believed to cover the source population of Danish seafarers and fishermen, we aimed to make a systematic overview of all disease groups by job title and time period, with estimation of standardized hospital contact ratios (SHCRs) using national rates, and to rank the disease groups by SHCR size. The results pertaining to skin and infectious diseases with sub-analyses are original to the present study, while results for other disease groups are partly derived from previous publications (38 out of a total of 184 SHCRs) in order to show the complete disease pattern among the three occupational groups and rank the SHCRs.

## 2. Experimental Section

### 2.1. General Information and Time Periods

The study population has been described in detail in three of our other papers (both genders in [7] and only males in [8,9]) and a short description adapted from these papers follows [7,8,9]. Two five-year follow-up studies, one starting on 1 January 1994 and the other on 1 January 1999, were conducted to examine potential changes over time in SHCRs of different diseases among Danish seafarers and fishermen. SHCRs were obtained by linking data from the Occupational Hospitalization Register (OHR) to occupational cohorts extracted from the almost complete data files with information on employment contracts for all seafarers and each ship they sign on and off in the Danish Seafarer Registry, administered by the Danish Maritime Authority (DMA) (seamen), and from the fishing boat yearbooks, fishery yearbooks, DMA files, tax registries, and pension registries (fishermen) [25,29]. For the fishermen, in particular, we combined several registries believed to cover all native Danish professional fishermen—fishing boat yearbooks, fishery yearbooks, the DMA files, and tax and pension registries—to identify all fishing boats in the Danish fishing industry operating from 1989 to 1998 and their company identification code [29]. This code was a means of extracting employee data from the ATP Registry, the largest national pension scheme, holding data on the occupational history of each person on board all the registered fishing boats. It is compulsory for all companies in Denmark whose employees are working nine hours or more per week to participate in a non-contributory pension scheme.

### 2.2. Occupational Hospitalization Register

The OHR contains information on each individual obtained through record linkage between three Danish national registers: the civil registration system (CRS), the Danish National Patient Registry (NPR), and the employment classification module [30]. In Denmark, hospital services are financed by taxes and for most of the population a hospital can be reached within 30–60 min or less. The NPR has existed since 1977 and contains data from all hospitals (more than 99% of all admissions) [31]. In the period 1977–1994, the registry only included inpatients, but since 1995 it comprised outpatients and emergency ward visits as well. The diagnoses have been coded according to the International Classification of Diseases, 10th revision (ICD-10) since 1994. The individual employment status is registered annually in the employment classification module [30]. The CRS contains information on gender, addresses, dates of birth, death and migration, and the unique PIN for every person who is or has been a Danish resident between 1968 and the present [32].

We used the PIN to link the occupational data with each fisherman’s or seafarer’s hospital contacts as an inpatient or outpatient as recorded by the nationwide OHR [30].

### 2.3. Inclusion Criteria for the Occupational Cohorts

Initially, we retrieved a total of 36,113 officers and non-officers of either gender (persons with and without residence in Denmark) and 11,755 male fishermen with a PIN and with records of employment aboard Danish ships during the period 1 January 1989 to 1 January 1999 from the files of the DMA and the pension register. Due to a high cohort turn-over, we restricted the cohorts to those employed on 1 January 1994 and 1 January 1999. As short-term employment is common, we restricted the definition of the occupational groups to at least six months of service (30.6% of the seafarers and 41.9% of the fishermen had a total service length in 1989–1998 of less than six months).

The inclusion criteria for follow-up of hospital contacts during 1994–1998 were thus: aged between 20 and 59 years on 1 January 1994, Danish residency according to the CRS, employment according to the employment classification module, and a total service as a seafarer or fisherman of at least six months in 1989–1993 according to the occupational registers. We used the same inclusion criteria for the follow-up of hospital contacts in 1999–2003, except that the starting date was 1 January 1999.

### 2.4. Follow-Up of Diagnoses in the Occupational Hospitalization Register

The PIN was used as a means of linking each person to the files of the nationwide OHR. The observation began either on 1 January 1994 or 1 January 1999. The follow-up ended on the date of diagnosis, date of death, date of emigration, or at the end of the study (31 December 1998 or 31 December 2003), whichever came first. We calculated the person-years (PY) at risk for each individual. Firstly, we made a follow-up and showed the time trend on the overall groups of ICD-10 diseases and chronic diseases. For further analyses, our focus was on more acute infectious diseases and on non-malignant skin diseases, because these diseases have been more rarely studied despite the fact that a number of previous studies of seafarers and fishermen have shown that these diseases may be associated with these occupations [19,24,25,26,27,28,33,34,35,36,37,38,39,40,41,42]. We selected the codes before the study started and included the ICD-10 codes: A00-B99, C00-D48, E00-E90, G00-G99, H00-H59, H60-H95, I00-I99, J00-J99, K00-K93, L00-L99, M00-M99, N00-N99, S00-T98, and A00-E90 + G00-T98.

### 2.5. Sub-Analyses

In sub-analyses of the main disease groups using a slightly different method, we retrieved information directly from the NPR (which provided data to the OHR) in order to make an extension of the follow-up period to 7 and 6.9 years, respectively, until 2000 and December 2005 [31]. However, no information was available about employment status in the reference group for these sub-analyses. Five referents “controls” for each seafarer were randomly selected among all persons of the same age, gender, and area of residency at the start of the studies at the baseline dates from the entire Danish population. The seafarers were eligible at the age of 20. For technical reasons, the randomly selected controls were matched on the birth date. Between birth and age 20 some controls had emigrated, died, or had one of the diagnoses under study, but these controls were not excluded in this supplementary sensitivity analyses.

### 2.6. Statistical Analyses

At the start of each follow-up period, the study participants were classified according to the type of job they held and the type of ship they worked on during their latest known employment in the five-year period preceding the baseline. The analyses were stratified by gender. The SHCR was calculated as 100 multiplied by the ratio between the observed and the expected number of cases. The latter was calculated by multiplying the PY at risk during the follow-up period in each five-year age and calendar year group by the corresponding rates of hospital contacts among economically active men or women in the total Danish population. The 95% confidence intervals (95% CI) of the SHCRs were estimated under the assumption that the observed number of cases followed a Poisson distribution. We calculated the exact intervals when the observed number of cases was less than 100. Otherwise, we used the propagation of error formulas and the central limit theorem to form a 95% CI around the logarithm of risk ratio, which we then transformed into a 95% CI around the risk ratio.

## 3. Results and Discussion

### 3.1. Study Population

The percentage of male seafarers with permanent residence in Denmark (as a percentage of all Danish and non-Danish seafarers on Danish ships) was 88% for officers, 90% for engineers, 77% for deck crew, 77% for engine crew, 75% for galley and catering crew, and 64% for other professions. For fishermen we were not able to make a separation on type of fishery or job title on board.

A total of 12,083 Danish seafarers and 4570 Danish fishermen were included at the start of the 1994 follow-up and 13,836 seafarers and 3470 fishermen at the start of the 1999follow-up (Table 1). Table 1 shows the number and characteristics of Danish fishermen and seafarers at the start of follow-up by gender and occupation.

**Table 1:** Danish fishermen and seafarers at baseline by gender and occupation **^1^**.

Characteristics	Job Type ^2^	Baseline 1994							Baseline 1999					
		Number	Mean Age ^3^	Vital Status					Number	Mean Age ^3^	Vital Status			
		N	Years	Alive ^4^		Emigrated ^5^			N	years	Alive ^4^		Emigrated ^5^	
				N	%	N	%				N	%	N	%
*Men*														
Fishermen	Fishermen	4570	37.2 ± 9.8	4468	97.8	58	1.3		3470	39.5 ± 10.1	3397	97.9	45	1.3
Seafarers	Officers	5061	40.2 ± 10.0	4942	97.7	167	3.3		5375	41.3 ± 10.2	5253	97.7	165	3.1
	Non-officers	5170	35.0 ± 11.1	5037	97.4	192	3.7		5867	37.2 ± 11.4	5731	97.7	171	2.9
*Women*														
Seafarers	Officers	90	32.8 ± 7.5	88	97.8	6	6.7		100	31.3 ± 7.3	100	100	4	4.0
	Non-officers	1762	31.6 ± 9.4	1752	99.4	53	3.0		2494	33.7 ± 10.5	2475	99.2	65	2.6

Notes: **^1^** Adapted from [7,8]. **^2^** Based on latest employment held before baseline. **^3^** Mean and standard deviation of age at baseline. **^4^** Number and percentage of people who were alive at the end of the follow-up period. **^5^** Number and percentage of people who emigrated during the follow-up period.

### 3.2. Standardized Hospital Contact Ratios for Overall ICD-10 Disease Groups by Gender, Job Type, and Time Period, with a Follow-up of Five or Seven Years

Table 2 and Table 3 show standardized incidence ratios (SHCR) for hospital contacts during 1994–2003 among Danish fishermen and seamen by job type and time period, for men and women, respectively. In total 38 out of the 150 SHCRs shown in Table 2 and Table 3 (38 out of a total of 184 SHCRs) are adapted from [7,8] to complete the total overview of hospital contacts among the three occupational groups, in order to make it possible to rank the size of the SHCRs for the specific ICD-10 disease groups [7,8].

**Table 2:** Standardized hospital contact ratios (SHCR) 1994–2003 among Danish fishermen and seamen by job type and time period (men) **^2^**.

ICD-10 ^1^	Site	Job Type	1994–1998			1999–2003			Trend ^2^	
			Cases	SHCR	95% CI	Cases	SHCR	95% CI	SHCR Ratio	95% CI
A00-B99	Infectious diseases	Fishermen	51	77	58–102	58	101	77–131	1.31	0.90–1.90
A00-B99		Officers	69	99	77–126	85	98	78–121	0.98	0.72–1.35
A00-B99		Non-officers	119	153	128–183	111	110	92–133	0.72	0.56–0.93
C00-D48	Neoplastic diseases	Fishermen ^3^	120	120	100–144	103	115	95–140	0.96	0.74–1.25
C00-D48		Officers ^3^	177	135	116–156	158	104	89–121	0.77	0.62–0.95
C00-D48		Non-officers ^3^	157	147	126–172	162	115	99–135	0.78	0.63–0.98
D50-D89	Lymphohematopoietic diseases	Fishermen	4	35	10–90	14	123	67–206	3.47	1.14–10.55
D50-D89		Officers	13	94	50–161	23	121	77–182	1.29	0.65–2.54
D50-D89		Non-officers	9	74	34–141	18	99	59–157	1.33	0.60–2.97
E00-E90	Endocrinal and nutritional diseases	Fishermen ^3^	40	67	48–92	44	82	59–110	1.21	0.79–1.86
E00-E90		Officers ^3^	71	97	76–122	98	111	90–135	1.15	0.84–1.55
E00-E90		Non-officers ^3^	82	135	107–167	102	124	102–150	0.92	0.69–1.23
G00-G99	Diseases of the nervous system	Fishermen	120	152	127–182	105	144	119–175	0.95	0.73–1.23
G00-G99		Officers	86	92	73–113	94	80	65–98	0.88	0.65–1.18
G00-G99		Non-officers	111	134	111–161	136	118	100–140	0.88	0.69–1.13
H00-H59	Diseases of the eye/ eye surroundings	Fishermen	62	65	50–84	55	62	47–81	0.95	0.66–1.37
H00-H59		Officers	63	59	45–75	107	77	64–93	1.31	0.96–1.78
H00-H59		Non-officers	104	96	79–116	152	103	87–120	1.07	0.83–1.37
H60-H95	Diseases of the ear/ proc mastoideus	Fishermen	71	87	68–109	78	101	80–126	1.16	0.84–1.60
H60-H95		Officers	97	91	73–110	124	93	78–111	1.03	0.79–1.34
H60-H95		Non-officers	69	78	61–99	125	101	85–121	1.29	0.96–1.73
I00-I99	Cardiovascular diseases	Fishermen ^3^	228	105	92–120	221	106	93–121	1.01	0.84–1.21
I00-I99		Officers ^3^	277	96	85–108	348	98	88–108	1.02	0.87–1.20
I00-I99		Non-officers ^3^	286	129	115–145	366	115	103–127	0.89	0.76–1.04
J00-J99	Respiratory diseases	Fishermen ^3^	150	105	89–123	129	111	94–132	1.06	0.84–1.35
J00-J99		Officers ^3^	149	93	79–109	154	85	73–99	0.91	0.73–1.14
J00-J99		Non-officers ^3^	194	118	103–136	225	114	100–130	0.97	0.80–1.17
K00-K93	Gastrointestinal diseases	Fishermen	385	125	113–138	299	114	102–128	0.91	0.78–1.06
K00-K93		Officers	354	96	87–107	385	91	82–101	0.94	0.82–1.09
K00-K93		Non-officers	414	125	114–138	508	121	111–132	0.97	0.85–1.10
L00-L99	Skin diseases incl. subcutaneous diseases	Fishermen	128	122	103–146	123	131	110–156	1.07	0.83–1.37
L00-L99		Officers	94	85	68–104	112	78	65–94	0.92	0.70–1.22
L00-L99		Non-officers	147	121	103–142	218	134	117–153	1.11	0.90–1.37
M00-M99	Bone, muscle, soft tissue diseases	Fishermen ^4^	546	122	113–133	494	118	108–129	0.97	0.86–1.09
M00-M99		Officers ^4^	384	75	68–83	499	75	69–82	1.00	0.87–1.14
M00-M99		Non-officers ^4^	530	107	98–117	730	106	98–114	0.99	0.88–1.11
N00-N99	Genitourinary tract diseases	Fishermen	104	76	63–92	99	81	66–99	1.07	0.81–1.41
N00-N99		Officers	160	98	84–114	229	118	104–135	1.21	0.99–1.48
N00-N99		Non-officers	149	100	85–117	215	108	94–123	1.08	0.88–1.33
S00-T98	Lesions to one or more body regions	Fishermen	1744	109	104–114	1487	118	112–124	1.08	1.01–1.16
S00-T98		Officers	1232	69	65–73	1471	69	66–73	1.00	0.93–1.08
S00-T98		Non-officers	2118	111	106–116	2584	114	109–118	1.02	0.96–1.08
A00-E90, G00-T98	All excl. F (psychiatric diseases)	Fishermen	2552	107	103–112	2084	113	108–118	1.05	0.99–1.11
A00-E90, G00-T98		Officers	2291	80	77–84	2610	80	77–83	1.00	0.94–1.06
A00-E90, G00-T98		Non-officers	3037	112	108–116	3568	113	109–116	1.01	0.96–1.06

Notes: **^1^** Classification ICD-10. **^2^** The trends (SHCR ratio) were only calculated if at least five cases were expected. **^3^** Results for total in the groups C00-D48, E00-E90, I00-I99, and J00-J99 adapted from [7]. **^4^** Results in the group M00-M99 adapted from [8].

**Table 3:** Standardized hospital contact ratios (SHCR) 1994–2003 among Danish seamen by job type and time period (women) **^2^**.

ICD-10 ^1^	Site	Job Type	1994–1998			1999–2003			Trend ^2^	
			Cases	SHCR	95% CI	Cases	SHCR	95% CI	SHCR ratio	95% CI
A00-B99	Infectious diseases	Officers	0	0	0–225	0	0	0–159	-	-
A00-B99		Non-officers	30	99	67–142	57	120	91–155	1.21	0.78–1.88
C00-D48	Neoplastic diseases	Officers	1	23	1–129	3	69	14–203	-	-
C00-D48		Non-officer ^3^	90	114	92–140	140	116	98–137	1.02	0.78–1.32
D50-D89	Lymphohematopoietic diseases	Officers	0	0	0–1171	0	0	0–752	-	-
D50-D89		Non-officers	4	81	22–209	9	86	40–164	-	-
E00-E90	Endocrinal and nutritional diseases	Officers	1	61	2–339	1	48	1–268	-	-
E00-E90		Non-officers ^3^	25	80	52–118	47	87	64–115	1.09	0.67–1.77
G00-G99	Diseases of the nervous system	Officers	3	188	39–548	0	0	0–146	-	-
G00-G99		Non-officers	37	120	85–165	58	110	84–143	0.92	0.61-1.39
H00-H59	Diseases of the eye/ eye surroundings	Officers	1	76	2–421	0	0	0–171	-	-
H00-H59		Non-officers	23	85	54–127	42	87	63–117	1.02	0.61–1.70
H60-H95	Diseases of the ear/ proc mastoideus	Officers	1	96	3–537	2	175	21–630	-	-
H60-H95		Non-officers	25	122	79–181	31	90	61–127	0.73	0.43–1.24
I00-I99	Cardiovascular diseases	Officers	1	34	1–189	4	106	29–272	-	-
I00-I99		Non-officers ^3^	73	135	106–170	124	118	99–140	0.87	0.65–1.16
J00-J99	Respiratory diseases	Officers	2	74	9–268	5	153	49–356	-	-
J00-J99		Non-officers ^3^	67	119	92–151	100	121	99–148	1.02	0.75-1.39
K00-K93	Gastrointestinal diseases	Officers	3	65	13–189	1	16	0–87	-	-
K00-K93		Non-officers	125	134	112–159	192	120	104–138	0.90	0.72–1.12
L00-L99	Skin diseases incl. subcutaneous diseases	Officers	3	140	29–410	2	65	8–235	-	-
L00-L99		Non-officers	50	111	82–146	94	125	101–153	1.12	0.80–1.59
M00-M99	Bone, muscle, soft tissue diseases	Officers	3	37	8–107	6	56	21–123	1.54	0.39–6.17
M00-M99		Non-officers	171	109	93–126	293	106	95–119	0.98	0.81–1.18
N00-N99	Genitourinary tract diseases	Officers	7	70	28–144	9	72	33–137	1.04	0.39–2.78
N00-N99		Non-officers	218	111	97–127	298	102	91–115	0.92	0.77–1.10
S00-T98	Lesions to one or more body regions	Officers	22	99	62–150	33	114	79–160	1.15	0.67–1.97
S00-T98		Non-officers	567	126	116–136	868	121	113–129	0.96	0.87–1.07
A00-E90, G00-T98	All excl. F (psychiatric diseases)	Officers	52	79	59–104	68	86	67–109	1.09	0.76–1.56
A00-E90, G00-T98		Non-officers	1277	110	104–116	1688	106	101–111	0.96	0.89–1.03

Notes: **^1^** Classification ICD-10. **^2^** The trends (SHCR ratio) were only calculated if at least five cases were expected. ^**3**^ Results for non-officers in the groups C00-D48, E00-E90, I00-I99, and J00-J99 adapted from [7].

For fishermen, the SHCRs for the ICD-10 disease group “all diseases combined (not including psychiatric diseases)” as well as for diseases of the nervous system, gastrointestinal diseases, skin diseases including subcutaneous diseases, bone, muscle, and soft tissue diseases, and lesions to one or more body regions exceeded the national rates in both time periods (Table 2). In 1994–1998 the highest SHCR was seen for diseases of the nervous system, followed by gastrointestinal and skin diseases, whereas in 1999–2003 it was for diseases of the nervous system followed by skin diseases and lymphohematopoietic diseases. The SHCRs for neoplastic diseases was statistically significantly elevated in the 1994 cohort, but not in the 1999 cohort of fishermen. Only skin diseases including subcutaneous diseases and lesions to one or more body regions showed both statistically significantly elevated SHCRs and increasing trends (statistically non-significant, however) between the two time periods.

Among male seafarers employed as non-officers, high SHCRs were observed in both time periods for the ICD-10 disease group “all diseases combined (not including psychiatric diseases)” as well as for endocrinal and nutritional diseases, diseases of the nervous system, cardiovascular diseases, respiratory diseases, gastrointestinal diseases, skin diseases including subcutaneous diseases, and lesions to one or more body regions. In 1994–1998 infectious diseases had the highest SHCR, followed by neoplastic and endocrinal diseases, whereas in 1999–2003, it was skin diseases followed by endocrinal and gastrointestinal diseases.

The SHCRs for the main infectious diseases group and neoplastic diseases were statistically significantly increased only in the 1994 cohort. For lymphohematopoietic diseases (as one group) the SHCRs were equivalent to those of the background population. Only skin diseases including subcutaneous diseases and genitourinary tract diseases showed both statistically significantly elevated SHCRs in 1999 and statistically non-significantly increasing trends between the two time periods.

Officers (navigation officers, radio officers, and engine officers) had a lower SHCR for the group “all diseases combined (not including psychiatric diseases)” than non-officers among seafarers, suggesting a social gradient in health. Officers’ SHCR for neoplastic diseases (as one group) was high in 1994 but close to that of the background population in the 1999 cohort. The SHCRs for genitourinary tract diseases was high only in the 1999 cohort. The trend for lympho-haematopoietic diseases, endocrinal and nutritional diseases, diseases of the eye/eye surroundings, and genitourinary tract diseases increased, however, between the two time periods.

Among women non-officer seafarers, high SHCRs were observed in both time periods for the ICD-10 disease group “all diseases combined (not including psychiatric diseases)” as well as for gastrointestinal diseases and lesions to one or more body regions in both cohorts, whereas the SHCRs for cardiovascular diseases were significantly increased only in the 1994 cohort, as was the SHCR for skin diseases including subcutaneous diseases in the 1999 cohort (Table 3). In 1994–1998 cardiovascular diseases had the highest SHCR, followed by gastrointestinal diseases and lesions, whereas in 1999–2003 it was skin diseases followed by lesions and respiratory diseases.

No SHCRs were found to be significantly lower than in the general population in either gender (Table 2 and Table 3).

Extending the follow-up period to seven years (until 31 December 2000 and 1 December 2005, respectively), using a slightly different method with a reference group matched to cases on birth year, gender, and area of residence at the baseline in 1994 and 1999, but without information on employment status in the reference group, showed similar results for the trend analyses (data not shown).

### 3.3. Standardized Hospital Contact Ratios for Infectious and Skin Diseases by Gender, Job Type, and Time Period

Overall, the SHCRs for infectious diseases among fishermen was not increased, but sub-analysis revealed high SHCR values for tuberculosis in both the 1994 and the 1999 cohorts (Table 4). The SHCR for all skin diseases combined was elevated in both time periods. We found high SHCR values for dermatitis and eczema among fishermen in the 1999 cohort and the risk of allergic dermatitis or eczema was high (SHCR 797 (95% CI: 472–1259)) based on 18 cases. The SHCR ratio for dermatitis showed a statistically significant relative increase between the two time periods.

Among Danish male officers, a statistically significantly increased SHCR was found in the 1994 cohort but not in the 1999 cohort for protozoal diseases (SHCR 783 (95% CI: 358–1486), based on eight cases). Among Danish male seafarers employed as non-officers, a high SHCR was observed for all infectious diseases combined in the 1994 cohort (SHCR 153 (95% CI 128–183)), but not in the 1999 cohort. The SHCR for HIV infections was statistically significantly increased in both the 1994 cohort and the 1999 cohort whereas the SHCRs for tuberculosis and hepatitis were statistically significantly increased only in the 1994 cohort.

Sub-analyses by type of ship in the 10-year follow-up period 1994–2003 showed that the highest SHCRs for hepatitis and HIV infection were found among male Danish seafarers working on dry cargo vessels including container ships, tankers, and coasters, and lowest on passenger ships (Table 5). Female seafarers working as non-officers had only a slightly (statistically non-significant) elevated SHCR for infectious diseases, and only in the 1999 cohort (Table 3).

Among non-officers, the SHCR for all skin diseases combined was statistically significantly increased in both the 1994 and the 1999 cohort for men, and in the 1999 cohort for women (Table 2 and Table 3). The SHCR for dermatitis or eczema for non-officers was 150 (95% CI: 101–206) for men and also 150 (95% CI: 90–234) for women (Table 4). The SHCRs for both allergic and non-specified eczema were elevated in the 1999 cohort for men, although allergic eczema fell short of statistical significance. Sub-analysis revealed a low SHCR value for eczema for engine room personnel (SHCR 59 (95% CI 26–117), based on eight cases).

**Table 4:** Standardized hospital contact ratios (SHCR) for infectious diseases and skin diseases, 1994–2003, among Danish fishermen as a whole; and among officers and non-officer seamen by time period.

ICD-10 ^1,2^	Site	Job Type	1994–1998			1999–2003			Trend ^3^	
			Cases	SHCR	95% CI	Cases	SHCR	95% CI	SHCR ratio	95% CI
*Men*										
A00-B99	Infectious diseases	Fishermen	51	77	58–102	58	101	77–131	1.31	0.90–1.90
A00-B99		Officers	69	99	77–126	85	98	78–121	0.98	0.72–1.35
A00-B99		Non-officers	119	153	128–183	111	110	92–133	0.72	0.56–0.93
A15-A19	Tuberculosis	Fishermen	6	446	164–971	9	954	437–1811		
A15-A19		Non-officers	5	349	113–814	4	252	69–645		
B15-B19	Hepatitis	Fishermen	3	83	17–242	4	150	41–385		
B15-B19		Non-officers	12	303	157–530	8	185	80–365		
B20-B24	HIV	Officers	4	143	39–366	3	146	30–426		
B20-B24		Non-officers	7	275	110–567	7	349	140–720		
B50-B54	Protozoal diseases	Officers	9	783	358–1486	3	249	51–726		
B50-B54		Non-officers	4	290	79–742	4	282	77–722		
Z20	Contact with infectious disease	Fishermen	10	241	116–443	8	110	47–217		
Z20		Officers	4	94	26–241	4	37	10–95		
Z20		Non-officers	8	166	72–327	12	97	50–170		
L00-L99	Skin diseases incl. subcutaneous diseases	Fishermen	128	122	103–146	123	131	110–156	1.07	0.83–1.37
L00-L99		Officers	94	85	68–104	112	78	65–94	0.92	0.70–1.22
L00-L99		Non-officers	147	121	103–142	218	134	117–153	1.11	0.90–1.37
L20-L30	Dermatitis and eczema	Fishermen	5	43	14–100	25	217	140–320	5.06	1.94–13.21
L20-L30		Officers	4	31	8–79	11	62	31–110	1.99	0.63–6.24
L20-L30		Non-officers	10	76	36–140	29	150	101–216	1.98	0.96–4.06
L23	Allergic dermatitis and eczema	Fishermen	3	153	32–447	18	797	472–1259		
L23		Non-officers	3	143	30–419	8	214	92–421		
L25	Non-specified contact dermatitis	Non-officers	1	73	2–405	4	289	79–740		
*Women*										
A00-B99	Infectious diseases	Non-officers	30	99	67–142	57	120	91–155	1.21	0.78–1.88
L00-L99	Skin diseases incl. subcutaneous diseases	Non-officers	50	111	82–146	94	125	101–153	1.12	0.80–1.59
L20-L30	Dermatitis and eczema	Non-officers	8	111	48–218	19	150	90–234	1.35	0.59–3.09
L23	Allergic dermatitis	Non-officers	1	57	2–315	3	97	20–283		
L24	Toxic dermatitis	Non-officers	3	246	51–720	3	165	34–483		

Notes: **^1^** Classification ICD-10. **^2^** Only diseases with at least three cases among at least one of the job types were shown. **^3^** The trends (SHCR ratio) were only calculated if at least five cases were expected.

**Table 5:** Standardized hospital contact ratios (SHCR) for infectious diseases in 1994–2003 among Danish seafarers by type of ship (men).

ICD-10 ^1,2^	Site	Type of Ship	Persons	Cases	SHCR	95% CI
A00-B99	Infectious diseases	Passenger	1992	72	123	96–155
A00-B99		Other	6495	243	127	112–144
A15-A19	Tuberculosis	Passenger	1992	3	262	54–767
A15-A19		Other	6495	7	195	78–402
B15-B19	Hepatitis	Passenger	1992	3	102	21–299
B15-B19		Other	6495	21	218	135–333
B20-B24	HIV	Passenger	1992	4	248	68–636
B20-B24		Other	6495	12	232	120–405

Notes: **^1^** Classification ICD-10. **^2^** Only diseases with at least three cases among at least one of the ship types were shown.

### 3.4. Discussion

#### 3.4.1. Main Findings

Surveillance of seafarers’ and fishermen’s health based on registries provides useful information about disease patterns over time in these occupational groups. We found a significantly increased overall relative risk of tuberculosis and dermatitis, especially allergic dermatitis, among fishermen. Among non-officers, the SHCR for HIV infection was statistically significantly increased in both the 1994 cohort and the 1999 cohort, whereas the SHCRs for tuberculosis and hepatitis were statistically significantly increased only in the 1994 cohort. The increased SHCR for hepatitis and HIV infection was only found among Danish seafarers working on ships other than passenger ships. The SHCRs for both allergic and non-specified eczema were elevated in the 1999 cohort for men.

#### 3.4.2. Main Strength and Possible Disadvantages of the Study

The main strength of the present study is that it is based on a comparison between two comprehensive cohorts of seafarers with a richness of employment details and a complete follow-up of health data using a nationwide register. Equally valid information was obtained for fishermen owing to the successful creation of a reliable fishermen cohort through a combination of several registries. The possible disadvantages of the study were a high turn-over of the cohort members, selection into the occupation as a seafarer or fisherman, the lack of data on seafarers of other nationalities than Danish, the fact that some groups (e.g., women) working as officers had few cases, and the fact that we had no access to data on specific exposures. For fishermen, the job title and type of fisheries were not available. Thus, the disease findings for fishermen reported in the present paper should be interpreted accordingly [43]. In the main analyses we used the economically active Danish population as a reference group in order to reduce the healthy worker effect, even though it is not expected to completely eliminate the problem. Selection of the cohort may still be a problem and the healthy worker effect may have been further enhanced by obligatory health examinations for seafarers [44]. We expect that our results are biased towards low estimates for some of the diseases studied and therefore did not present the results of the seven-year follow-up without information about occupational status for the controls as the main finding.

Referral bias may constitute a problem in studies using hospital admissions as a measure of disease, especially for diseases commonly treated outside hospitals (e.g., skin diseases) and diseases for which people are only admitted to hospital if it progresses to a severe disease [30]. Admission of seafarers and fishermen to hospitals in other countries may take place for acute diseases and accidents, so low risks are therefore not necessarily interpretable. Hospitalization abroad that is not followed by hospitalization in Denmark will bias risk estimate towards null, but we expect the majority of seafarers and fishermen with severe chronic or protracted diseases to be followed up in a Danish hospital. Similarly, we expect misclassification of diseases due to possible coding errors in the registries to be non-differential, with a similar proportion of errors among seafarers, fishermen, and the Danish working population in general. Three Danish studies have formerly estimated predictive positive values (PPV) of infections recorded in the NPR compared to [45,46,47]. A study from 1998 found a low PPV for septicemia compared to a database of positive blood cultures [45]. In contrast, a high PPV was seen for the codes of the diagnoses of patients with pneumonia hospitalized in 1994–2003, and a recent study revealed a high PPV of 98% of primary inpatient diagnosis of infection among cancer patients in 2006–2010 in the NPR compared to medical charts, a PPV of 77% for specific types of infections; for skin infections, pneumonia, and sepsis, PPVs were 79%, 93%, and 84%, respectively [46,47].

#### 3.4.3. Comparison with Other Studies

We compared SHCRs for all disease groups in two five-year follow-up time periods (until 1999 and until 2003), in sub-analyses in seven-year time periods (until 2005), and in analyses of infections extending the follow-up period to 10 years, and found rather similar results. The results for the large groups of mainly lifestyle-related chronic diseases were similar to the previous literature, of which [7,8,9] include our own results, [8,9] for men only [5,6,7,8,9]. Whether the proper follow-up time should be five, seven or even 10 years probably depends on the disease group under study, with the chronic diseases probably requiring a rather long follow-up time of five years or more. On the other hand, a shorter follow-up time may be more appropriate for primary acute conditions like injuries, to diminish the risk of the seafarer or fisherman having acquired the disease under study many years after he or she had left the trade. Taking account of periods with unemployment and employment in other trades, as well as of specific exposures, may give an even more accurate estimate of the SHCR for acute conditions such as injuries within the trade as seafarers and fishermen.

The literature points to an increased risk among seafarers and fishermen of dermatoses either caused by marine organisms or by chemical exposures such as spills of polychlorinated biphenyls (PCB) containing oils or after injuries involving other chemicals carried either as cargo or as needed on the ship, or cutaneous cancers, fish bites or stings, and infections [38,39,40,41,42]. However, cutaneous exposure to oils does not seem to be the main cause of the increased occurrence of skin diseases among seafarers. Both male fishermen and non-officer seamen have increased SHCRs for skin diseases in general, but the SHCR for eczema specifically among engine room personnel was low (59, 95% CI 26–117). Working in a wet environment, combined with UV-induced skin lesions (or, for fishermen also, dermatoses caused by marine organisms or machine scratches) is probably the main cause of skin problems among these occupational groups.

The elevated SHCR for incident HIV infection among non-officers in the merchant fleet has not declined in 2003 or 2005 despite information campaigns regarding sexual behavior abroad [24,25,26,35]. Just as in the literature, jobs with an increased morbidity of HIV—here, non-officers—also had more cases of viral hepatitis than expected [13]. Sub-analyses by type of ship in the 10-year follow-up period 1994–2003 showed that the highest SHCRs for hepatitis and HIV infection were found among male Danish seafarers working on dry cargo vessels including container ships, tankers, and coasters, and lowest on passenger ships. The elevated tuberculosis level among fishermen based on a few cases may probably be due to infection on shore as previously shown for non-officer seafarers (based on bacterial DNA finger printing) in a Danish study [37]. This study from 2006 identified the most likely route of M. tuberculosis transmission among seafarers to be infection ashore—for the majority of them, a long time after they had stopped working within the trade. The strains of tuberculosis identified were similar to those seen mainly among socially marginalized subgroups of people ashore characterized by alcohol abuse. Only a few seemed infected by voyages to countries with a high incidence of tuberculosis or ships with international crews from high-incidence countries. Similarly, the high risk of hepatitis and HIV seen in our study may also be caused by close contact with certain high risk groups in Denmark, where these infections are more frequent.

## 4. Conclusions

In conclusion, using a combination of administrative registries gives useful information on Danish seafarers’ and fishermen’s health and showed increased SHCRs for a number of chronic diseases and lesions and an increased trend for certain infectious and skin diseases. We found high SHCR values for tuberculosis and dermatitis, especially allergic dermatitis, among fishermen. Both male fishermen and non-officer seamen have increased SHCRs for skin diseases in general, but the SHCR for eczema, specifically among engine room personnel, was low, suggesting mainly other causes than oil exposure. Among non-officers, the SHCR for HIV infection was statistically significantly increased in both the 1994 cohort and the 1999 cohort, whereas the SHCRs for tuberculosis and hepatitis were statistically significantly increased only in the 1994 cohort. The increased SHCRs for hepatitis and HIV infection were solely found among seafarers working on ships other than passenger ships. The SHCR for dermatitis was elevated in the 1999 cohort for men working as non-officers. The suggested model for disease surveillance among seafarers and fishermen is recommended to be regularly updated with new analyses every five years and with five, seven or 10 years of follow-up time, depending of the nature of the diseases under study.
